# The Identification of Precursor Regulation Impact on the Methamphetamine Market and Public Health Indicators in the Czech Republic: Time Series Structural Break Analysis

**DOI:** 10.3390/ijerph17217840

**Published:** 2020-10-26

**Authors:** Benjamin Petruželka, Miroslav Barták

**Affiliations:** Department of Addictology, First Faculty of Medicine, Charles University and General University Hospital, 128 00 Prague, Czech Republic; miroslav.bartak@lf1.cuni.cz

**Keywords:** methamphetamine, precursor, regulation, pseudoephedrine, Czech Republic, drug supply, drug market, time series, nonfatal intoxication, public health

## Abstract

Background: This study provides insight into the impact of methamphetamine precursor regulation, which is considered to be one of the most important tools of supply reduction and a tool with potential public health impact. Methods: It is based on a longitudinal and quasi-experimental design and it investigates the changes of methamphetamine precursor regulation in Czech Republic, which is treated as a natural experiment. The statistical analysis uses features from the generalized fluctuation test framework as well as from the F test framework to estimate structural changes in the methamphetamine-related arrests and nonfatal intoxications time series. Results: The analysis identified structural breaks in the majority of the methamphetamine drug market-related time series in the period related to the tightening of regulation. The results of this study show that methamphetamine precursor regulation was associated with the proliferation of international and organized crime groups and with no change in the overall number of arrests and nonfatal intoxications. Conclusions: The precursor regulation ceteris paribus plausibly leads to the change in drug supply towards more organized groups and to an increasing involvement of foreign nationals at the drug market and is not effective in suppressing the methamphetamine market and in reducing the public health indicator of nonfatal methamphetamine intoxications.

## 1. Introduction

At the global level, methamphetamine is a changing and increasing issue in many countries [1] and the quantity of seized methamphetamine is also increasing [2]. The use of methamphetamine poses a significant public health problem because it causes major physical and psychological harms [3] and individual and societal costs [4,5]. The characteristics of methamphetamine production condition the possibilities of law enforcement and other regulation efforts to reduce the supply of methamphetamine. The production of methamphetamine is characterized by the diversion of the chemicals from the pharmaceutical and chemical industries [6]. If available, producers of methamphetamine use readily available chemicals that make the production relatively easy [7]. From the global perspective, precursors such as ephedrine or pseudoephedrine are cheap and available because they are widely and legally used, especially in the pharmaceutical industry [8].

International Narcotics Control Board [9] suggests that control of the above described intersection between legitimate and illegitimate business is one of the most valuable tools of supply reduction. Therefore, it might be considered as an important public health tool, which aims to influence a public health issue by altering the environment. The regulation of this intersection at the methamphetamine market was tightened at the turn of the 21st century. The tightening of regulation of medicines containing pseudoephedrine was described over the globe, for instance in the USA [10], Australia [11], New Zealand [12], and Turkey [13]. The effectiveness of different methamphetamine regulations was systematically reviewed and it was found that in general, some regulations were effective in reducing methamphetamine drug markets, use, and harms while others were not [7]. The ineffectiveness was attributed to the existence of alternative sources of precursors or the availability of imported methamphetamine. However, according to this review, the regulations of methamphetamine precursors were examined only in North America. This is problematic because we ought to know how methamphetamine regulation works in other contexts. The influence of the regulation has recently been investigated in Australia [14].

Studies focusing on the impact of methamphetamine precursor regulations on methamphetamine market and supply have found different effects. Cunningham and Liu [15] found out that regulation targeting precursors in the USA used by small-scale producers had no impact on methamphetamine arrests, but regulations targeting precursor chemicals used by large-scale producers had an impact, lowering the number of arrests; however, they also found out that the arrests have rebounded. Dobkin and Nicosia [16] and Dobkin et al. [17] focused on different methamphetamine precursor regulations in the USA. Dobkin and Nicosia [16] found out that although the methamphetamine arrests fell by 50%, the impact was largely temporary and arrests approached preintervention levels within 18 months. Dobkin et al. [17] found no evidence of changes in arrests for drug possession and suggested people were able to find methamphetamine produced elsewhere with production probably shifted across national borders. Mazerolle et al. [14] found out that in Australia precursor regulation (pharmaceutical electronic tracking systems) was associated with a decrease in the production of methamphetamine, increase in supply incidents, and no change in possession. Based on this, Mazerolle et al. [14] concluded that this regulation can reduce domestic production but does not impact possession, distribution, and importation. The impact of methamphetamine regulation on methamphetamine market and supply might be influenced by the import of precursors from neighboring countries, undermining the regulation [7,17]. In the case of the USA regulations methamphetamine was imported from Mexico [17,18]. It was suggested that with tougher regulation, Mexican organizations began to enter the US drug market [18,19]. It is plausible that the regulation having an impact on the drug market and availability of the drugs should also influence the public health impacts of the drug market. However, the research showed different results. Callaghan’s et al. [20] study from Canada indicates that some regulations of methamphetamine precursors were not related to the changes in methamphetamine-related hospital admissions and some were related to an increase thereof. The increase is explained by the shift in methamphetamine production from small/scale to criminal organizations better able to circumvent the regulation. It was also found that the regulation focused on small-scale producers had little effect [21]. On the contrary, the studies conducted by Cunningham and Liu [21] and Dobkin and Nicosia [16] showed a decrease in hospital admissions.

In Europe, the issue of methamphetamine has raised some concerns. However, based on available evidence, it might be concluded that methamphetamine use is not a major phenomenon across all of Europe [22,23,24]. Historically, the use and production of methamphetamine in Europe has largely been confined to the Czech Republic and Slovakia [23]. Its emergence dating back to the 1970s, the market for methamphetamine in the Czech Republic is well-established [25]. Currently, treatment entrants reports in the Czech Republic and Slovakia account for almost 90% of methamphetamine users in Europe [26]. Of the 300 illicit methamphetamine laboratories reported in the European Union in 2017, 264 were found in the Czech Republic [24].

In central Europe, methamphetamine is usually made from pseudoephedrine, which is extracted from diverted medications used as cold or flu remedies [24,26,27,28]. Ephedrine and pseudoephedrine are internationally controlled drug precursors. They are the main precursors used in the production of methamphetamine in the Czech Republic, Germany, Poland, Slovakia and a number of other member States of the European Union [24]. During the entire period of the study 2004–2016, the main source for the production of methamphetamine in the Czech Republic was medications containing pseudoephedrine [29]. Starting in 2009, the regulation of these medications underwent rapid changes in the Czech Republic and was gradually tightened (for an overview, see Petruželka [29]). In 2007–2008, these medications were usually sourced locally and in pharmacies. This changed with the tightening of regulation, following which these medications were sourced from other and primarily neighboring countries [29]. It has been suggested that the change in the source was accompanied by a gradual process, which has been ongoing since the regulation and culminated around 2014, of involvement of organized crime groups, foreign nationals (Vietnam and neighboring countries) in the Czech methamphetamine market and emergence of industrial-like labs in a market previously dominated by small-scale “kitchen” labs [29]. This period might have been influenced by other factors than the regulation of the precursors. However, the previous analysis identified as the only significant factor change in the police priorities in 2013. In this year, the official documents included drug market policing as one of the priorities and it plausibly increased the overall number of arrests that was associated to increase nonfatal intoxications and infectious diseases such as hepatitis C [30,31,32]. In comparison to the previous research focused on change in police priorities and its impact on public health indicators [32], this article focuses on different outcome variables (overall number of arrests, involvement of organized crime groups and foreign nationals), uses a different method (structural break analysis compared to intervention time series analysis), and focuses analytically also on the previous period instead of the narrow focus on the impact of changes in 2013.

This study uses a quasi-experimental research design to examine the methamphetamine precursor regulation impact on the methamphetamine drug market (the overall rate of methamphetamine and precursor-related arrests and involvement of organized crime groups and foreign nationals) and on public health (nonfatal methamphetamine intoxications). The advantage of this study is that it provides a statistical analysis of the impact of methamphetamine precursor regulation, which has previously only been described based on official reports, see Petruželka [29]. Furthermore, this study complements other studies from the North American and Australian contexts, and demonstrates how methamphetamine precursor regulation works at the well-established methamphetamine market in the Czech Republic. The design includes methods based on dating structural changes in regression models including time, using features from the generalized fluctuation test framework as well as from the F test framework [33]. The advantage of this approach is that the date of change is identified endogenously (only from the data) and not by the researcher. In the case of changes in methamphetamine precursor regulation in the Czech Republic, this approach is beneficial because the changes were gradual, took over a year, and the effect of some of these changes was plausibly delayed, cf. Petruželka [29] for the summary of the changes, and therefore there was no specific date of intervention for the time series intervention analysis. The data for the analysis are based on the statistics collected by the National Drug Headquarters of the Criminal Police and Investigation Service (NDH) and the Institute of Health Information and Statistics of the Czech Republic (IHIS CR) in the period of 2004–2016.

## 2. Materials and Methods

### 2.1. Design

We used a longitudinal and quasi-experimental design based on the changes in methamphetamine precursor regulation in the Czech Republic, which is treated as a natural experiment. The analysis was conducted for the years 2004–2016 using monthly aggregation of the data.

We estimated structural changes in the methamphetamine, precursor, and heroin-related arrests time series and methamphetamine and heroin-related time series. Our method is based on dating structural changes in the regression model including time, using features from the generalized fluctuation test framework as well as from the F test framework [33]. This approach identifies whether the regression coefficients remain constant against the alternative that at least one coefficient varies over time [34]. This approach was fit for the analysis because it allowed us to find the changes from the data endogenously. The changes in the regulation took over a year and the effect of some of these changes was plausibly delayed, cf. [29] and thus there was no specific date of intervention for time series intervention analysis [35].

We employed a simpler approach for the control: a separate analysis of the variable [35]. We analyzed the changes in the heroin time series separately to see if there were any other underlying factors that might have influenced the time series. The heroin control series was used in a similar manner by Cunningham and Liu [15] in their analysis of methamphetamine regulation influence on methamphetamine arrests.

### 2.2. Data and Its Availability and Collectability

We used data collected by the National Drug Headquarters providing the information about individual arrests. The advantage of this data set is its detail and specificity, considering the illegal substances, precursors, and legal qualification. However, data for the year 2009 were missing. These data were imputed using the imputeTS package [36]. The imputeTS package was used to impute the missing data for drug-related arrest time series. We used the Kalman Smoothing and Simple or Exponential Moving Average according to the characteristics of time series in line with procedures described by [36,37]. Furthermore, we used data collected by IHIS CR providing information about individual cases of methamphetamine related nonfatal intoxications from the NRHOSP (Národní registr hospitalizovaných) registry, which records only cases requiring hospitalization for more than 24 h. The contract between one of the researchers and the institutions does not allow the publication of the original data.

### 2.3. Variables

Considering the arrest data, we constructed monthly aggregated time series based on the legal qualification, on information about the substances involved in the arrest and on information about the arrested. The information about the legal qualification as defined in the penal code and used by the police for classification of the arrests was used to categorize arrests related to organized crime groups. The penal code changed in 2010, however, this did not bring significant changes for the drug-related organized crime qualification because the facts that constitute a crime in this area remained nearly the same (former penal code: § 187, 2a, § 187, 4c, current penal code: § 283/2a, § 283/4c) except the new qualification (actual penal code § 286, 2a), cf. [38]. However, this new qualification was not used in the data set in relation to the methamphetamine and precursor-related arrests. The information about citizenship of the arrested was used to attribute the nationality to the arrest.

We produced two basic categories of time series. The methamphetamine-related time series and control time series. The methamphetamine-related time series had three subcategories: methamphetamine, precursors, and organized crime (including methamphetamine and precursors). The first subcategory included arrests that were only related to methamphetamine, the arrests related to methamphetamine precursors were not included. The second subcategory included only arrests related to methamphetamine precursors, the arrests related only to methamphetamine were not included. The third subcategory (organized crime) included both methamphetamine and precursor-related arrests because the number of organized crime cases was rather low. The control time series had two subcategories, heroin and organized crime heroin arrests. In all these subcategories, we produced the following time series: overall number of arrests, arrests of foreign (all other nationals excluding the Czech), Vietnamese, Czech, and neighboring countries nationals (Slovakia, Poland, Austria and Germany).

We constructed two variables of nonfatal intoxications from the data set provided by the IHIS CR. One was related to methamphetamine and the other one to heroin. The ICD-10 categories were used to identify cases related to both categories. The cases of accidental, intentional, or undetermined poisoning caused by illegal drugs were extracted, i.e., diagnoses of intoxications with psychostimulants (T43.6) and heroin (T40.1). The category of psychostimulants plausibly contains the cases of methamphetamine overdoses, since methamphetamine is historically the main psychostimulant and drug among high-risk drug users in the Czech Republic and it is used as a measure for methamphetamine intoxications in public health reports [22,39].

### 2.4. Statistical Analysis

The analysis was conducted in R software, using the strucchange package [33] and imputeTS package [36]. The statistical analysis of data was conducted using the strucchange package and in line with the Zeileis et al. [34] procedure. The constant was fitted to the data, and using OLS-based CUSUM process and tests based on F statistics it was tested whether the mean of the time series (in different categories) changes over time. The strucchange package (RSS and BIC values) was used to identify the breakpoints and their confidence intervals only if the change was identified in the time series. The information about the breakpoints was used to fit the linear models with different segments (before and after breakpoints) and to calculate intercepts (monthly means) in different segments.

## 3. Results

The missing data for drug-related arrests were imputed, for results see Appendix A (Figure A1, Figure A2, Figure A3, Figure A4, Figure A5, Figure A6, Figure A7, Figure A8, Figure A9, Figure A10, Figure A11, Figure A12, Figure A13, Figure A14, Figure A15, Figure A16, Figure A17, Figure A18, Figure A19, Figure A20, Figure A21, Figure A22, Figure A23, Figure A24 and Figure A25). The statistical analysis identified structural breaks in a number of the time series, i.e., the time series did not exhibit constant model coefficients (see Table 1 and Appendix B, Figure A26, Figure A27, Figure A28, Figure A29, Figure A30, Figure A31, Figure A32, Figure A33, Figure A34, Figure A35, Figure A36, Figure A37, Figure A38, Figure A39, Figure A40, Figure A41, Figure A42, Figure A43, Figure A44, Figure A45, Figure A46, Figure A47, Figure A48, Figure A49, Figure A50, Figure A51 and Figure A52). The time series with a structural break included one or more of them.

The structural breaks were identified in all nonfatal intoxications-related time series (see Table 1 and Appendix B, Figure A51 and Figure A52). The methamphetamine time series had one breakpoint in the 11th month of 2013 (cf. 2012/12, 2014/4) and heroin time series in the 7th month of 2010 (cf. 2010/2, 2011/11). The intercept of methamphetamine time series was higher in the second than in the previous segment, while the intercept of heroin time series was lower in the second than in the previous segments (see Table 2).

**Figure A26:**
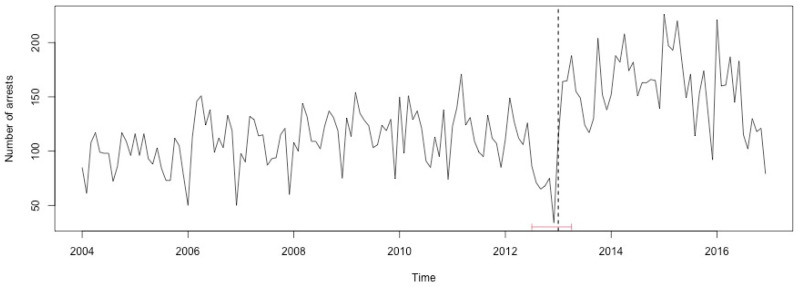
Methamphetamine arrests 2004–2016 in the Czech Republic and structural breaks.

**Figure A27:**
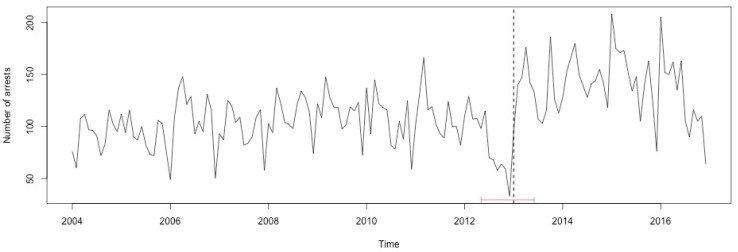
Methamphetamine Czech nationals arrests 2004–2016 in the Czech Republic and structural breaks.

**Figure A28:**
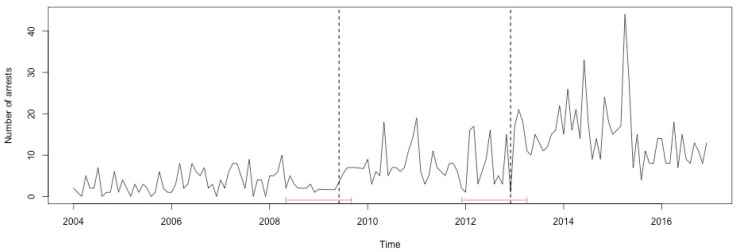
Methamphetamine foreign nationals arrests 2004–2016 in the Czech Republic and structural breaks.

**Figure A29:**
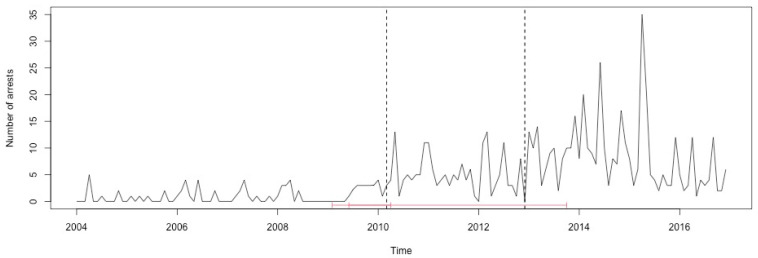
Methamphetamine Vietnamese nationals arrests 2004–2016 in the Czech Republic and structural breaks.

**Figure A30:**
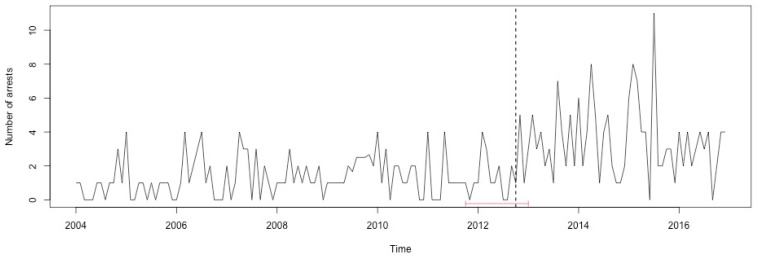
Methamphetamine neighboring countries nationals arrests 2004–2016 in the Czech Republic and structural breaks.

The structural breaks were identified in all methamphetamine arrests time series (see Table 1 and Appendix B, Figure A26, Figure A27, Figure A28, Figure A29 and Figure A30). The intercepts in different segments in all methamphetamine time series were higher than in previous segments (see Table 2). The overall methamphetamine time series had second breakpoint in the 2nd month of 2013 (cf. 2012/8, 2013/5), Czech nationals one breakpoint time series in the 2nd month of 2013 (cf. 2012/8, 2013/7), foreign nationals time series two breakpoints: the 6th month of 2009 (cf. 2008/5, 2009/9) and the 1st month of 2013 (cf. 2011/12, 2013/4); Vietnamese nationals time series two breakpoints: the 3rd month of 2010 (cf. 2009/6, 2010/4) and the 12th month of 2012 (cf. 2009/3, 2013/11), neighboring countries nationals one breakpoint in time series in the 10th month 2012 (cf. 2011/10, 2013/1).

**Figure A31:**
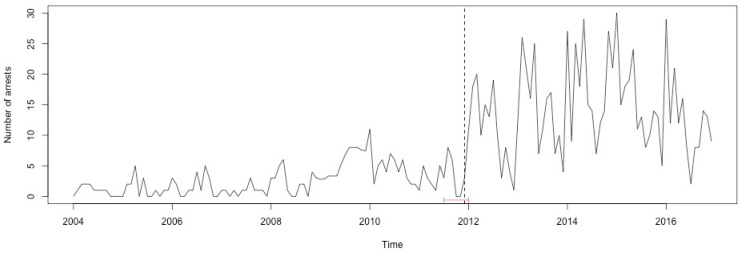
Precursor arrests 2004–2016 in the Czech Republic and structural breaks.

**Figure A32:**
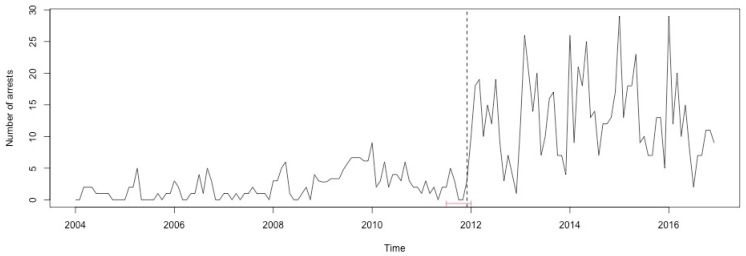
Precursor Czech nationals arrests 2004–2016 in the Czech Republic and structural breaks.

**Figure A33:**
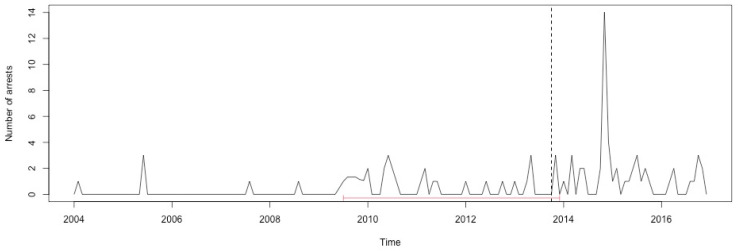
Precursor foreign nationals arrests 2004–2016 in the Czech Republic and structural breaks.

**Figure A34:**
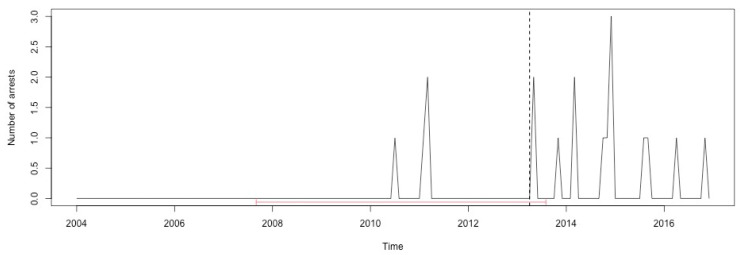
Precursor Vietnamese arrests 2004–2016 in the Czech Republic and structural breaks.

**Figure A35:**
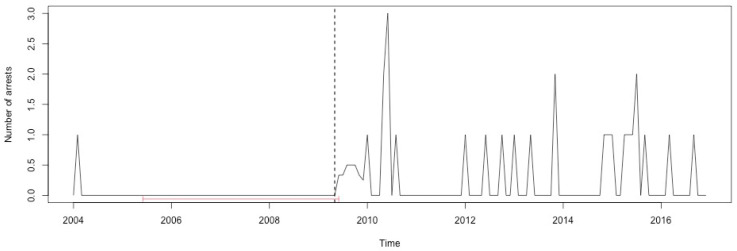
Precursor neighboring countries arrests 2004–2016 in the Czech Republic and structural breaks.

In each precursor time series one structural break was identified (see Table 1 and Appendix B, Figure A31, Figure A32, Figure A33, Figure A34 and Figure A35) and the intercepts in different segments in all models were higher than in the previous segments (see Table 2). Structural breaks in the period before 2010 were identified only in Czech and Vietnamese nationals time series. The precursor time series had the following structural breaks: the overall time series in the 12th month of 2011 (cf. 2011/7, 2012/1), Czech nationals in the 12th month of 2011 (cf. 2011/7, 2012/1), foreign nationals in the 10th month of 2013 (cf. 2009/8, 2013/12), Vietnamese nationals in the 4th month of 2013 (cf. 2007/9, 2013/8), neighboring countries in the 5th month of 2009 (cf. 2005/6, 2009/6).

**Figure A36:**
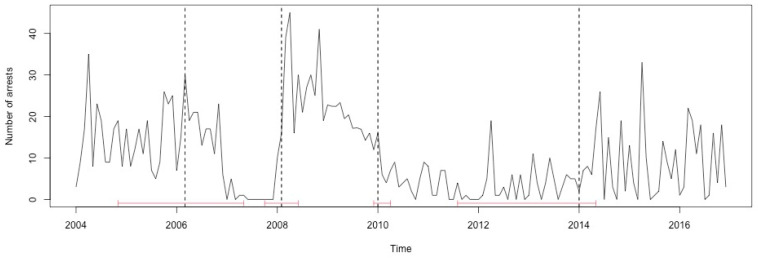
Methamphetamine-related organized crime arrests 2004–2016 in the Czech Republic and structural breaks.

**Figure A37:**
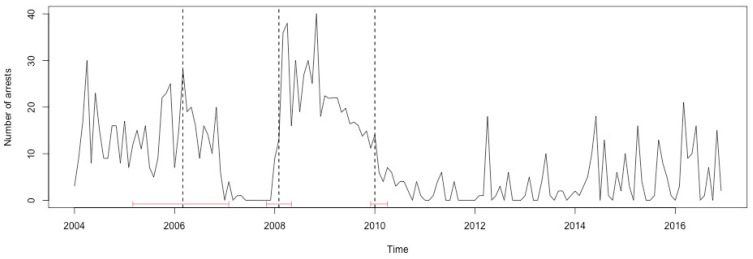
Methamphetamine-related Czech nationals organized crime arrests 2004–2016 in the Czech Republic and structural breaks.

**Figure A38:**
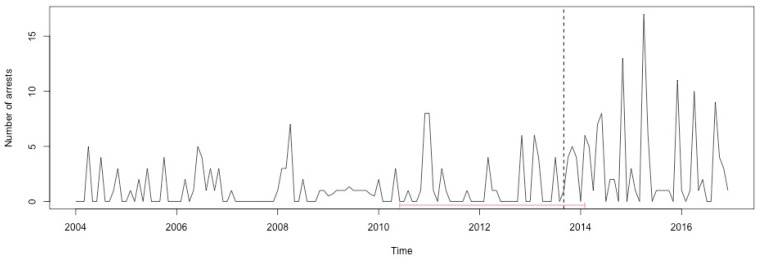
Methamphetamine-related foreign nationals organized crime arrests 2004–2016 in the Czech Republic and structural breaks.

**Figure A39:**
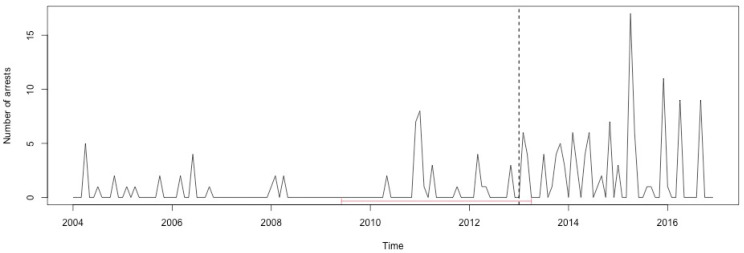
Methamphetamine-related Vietnamese nationals organized crime arrests 2004–2016 in the Czech Republic and structural breaks.

**Figure A40:**
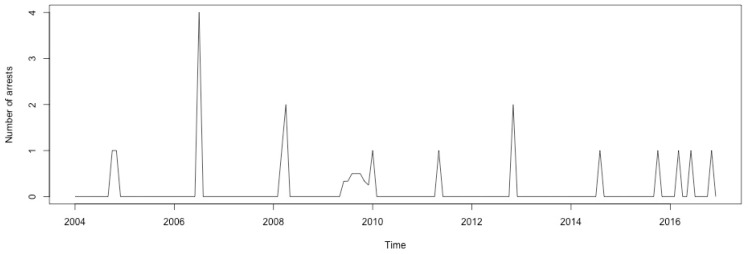
Methamphetamine-related neighboring countries nationals organized crime arrests 2004–2016 in the Czech Republic and structural breaks.

Structural breaks were identified in all methamphetamine-related organized crime time series except the neighboring countries nationals (see Table 1 and Appendix B, Figure A36, Figure A37, Figure A38, Figure A39 and Figure A40). The methamphetamine-related organized crime time series is divided into five segments by four breakpoints (see Table 1 and Appendix B, Figure A36), which show an increase and decrease in the value of intercepts (see Table 2). The methamphetamine-related Czech nationals organized crime time series is divided into four segments by four breakpoints (see Table 1 and Appendix B, Figure A37), which show an increase and decrease in the value of intercepts (see Table 2). One structural break was identified in the foreign nationals (2013/9, cf. 2010/6, 2014/2) and Vietnamese national time series (2013/1, cf. 2009/6, 2013/4) and it was characterized by an increase in the value of intercepts.

**Figure A41:**
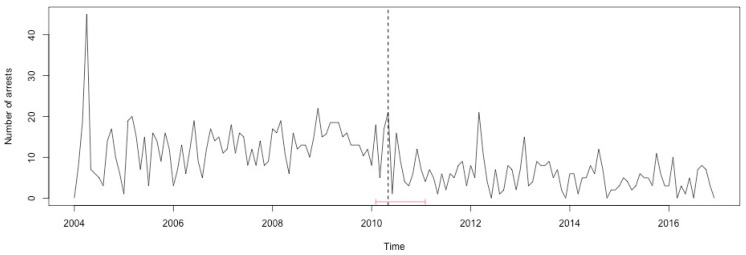
Heroin arrests 2004–2016 in the Czech Republic and structural breaks.

**Figure A42:**
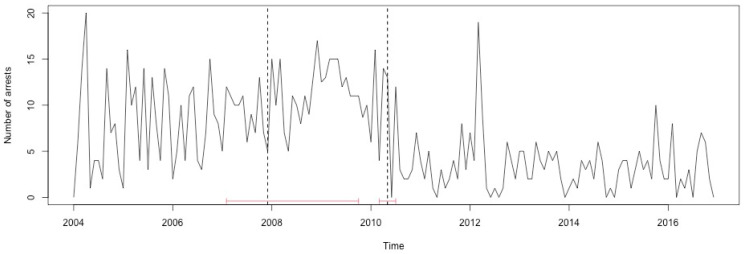
Heroin Czech nationals arrests 2004–2016 in the Czech Republic and structural breaks.

**Figure A43:**
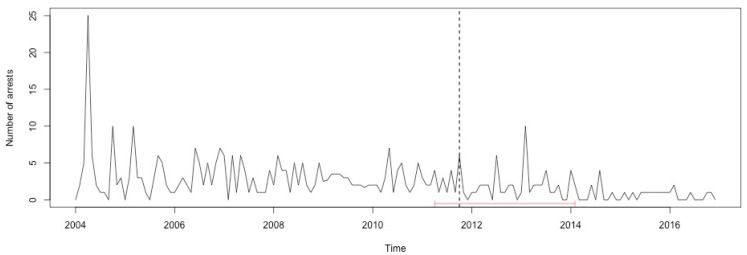
Heroin foreign nationals arrests 2004–2016 in the Czech Republic and structural breaks.

**Figure A44:**
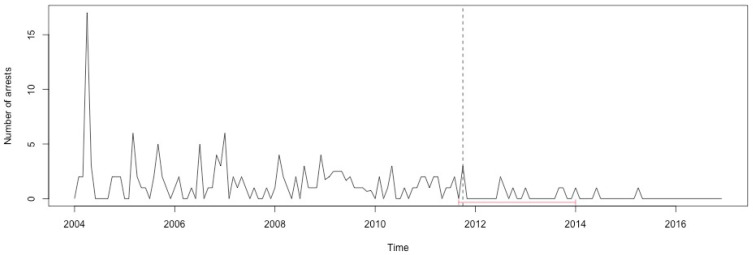
Heroin Vietnamese nationals arrests 2004–2016 in the Czech Republic and structural breaks.

**Figure A45:**
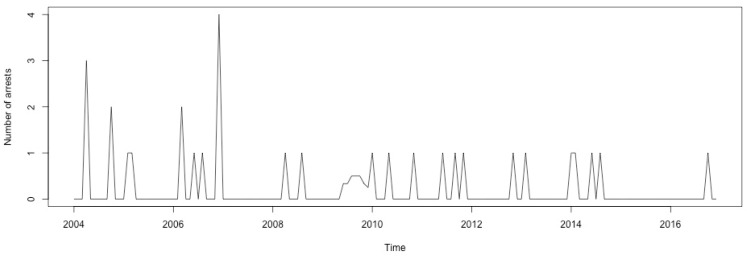
Heroin neighboring countries nationals arrests 2004–2016 in the Czech Republic and structural breaks.

**Figure A46:**
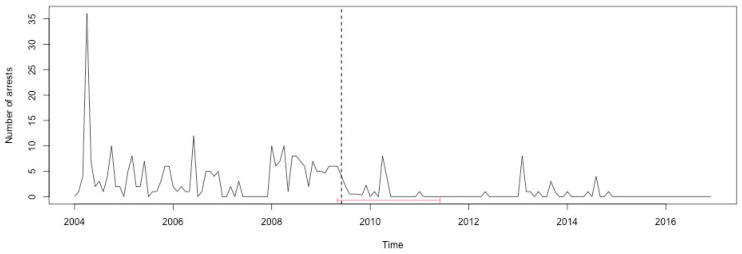
Heroin organized crime arrests 2004–2016 in the Czech Republic and structural breaks.

**Figure A47:**
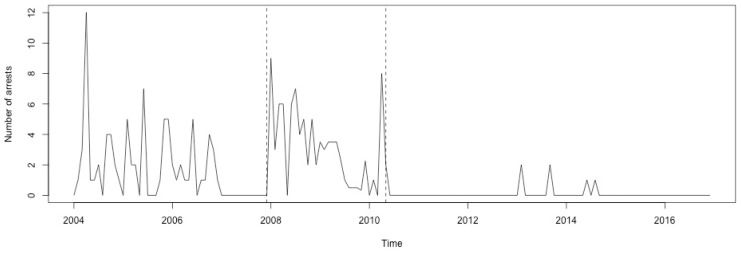
Heroin organized crime Czech nationals arrests 2004–2016 in the Czech Republic and structural breaks.

**Figure A48:**
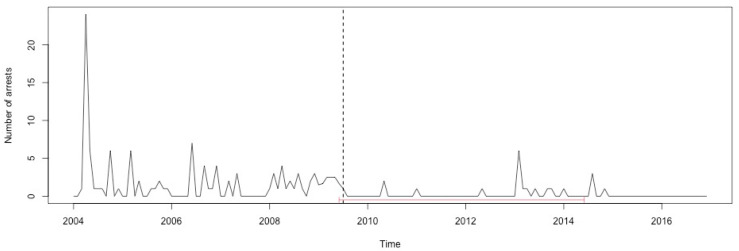
Heroin organized crime foreign nationals arrests 2004–2016 in the Czech Republic and structural breaks.

**Figure A49:**
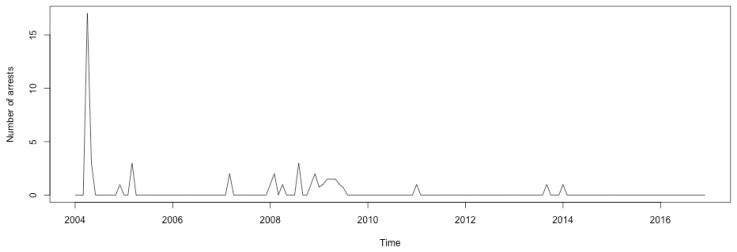
Heroin organized crime Vietnamese nationals arrests 2004–2016 in the Czech Republic and structural breaks.

**Figure A50:**
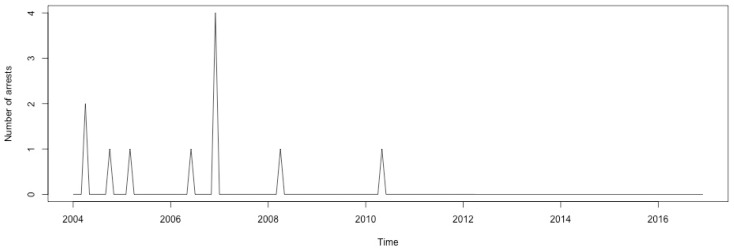
Heroin organized crime neighboring countries nationals arrests 2004–2016 in the Czech Republic and structural breaks.

**Figure A51:**
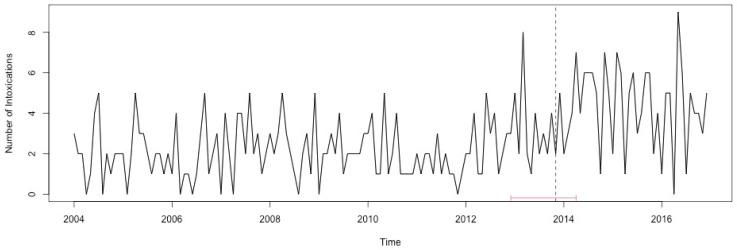
Methamphetamine intoxications 2004–2016 in the Czech Republic and structural breaks.

**Figure A52:**
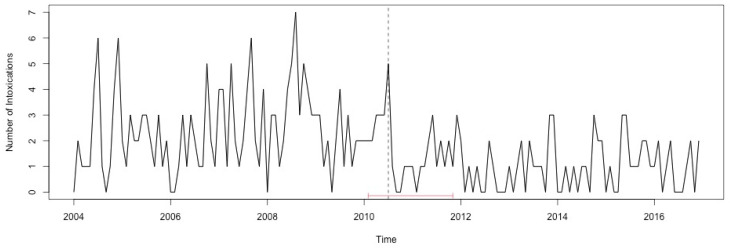
Heroin intoxications 2004–2016 in the Czech Republic and structural breaks.

Structural breaks were identified in all control heroin and organized crime heroin-related time series except the neighboring countries and Vietnamese nationals time series (see Table 1 and Appendix B, Figure A41, Figure A42, Figure A43, Figure A44, Figure A45, Figure A46, Figure A47, Figure A48, Figure A49 and Figure A50). The intercepts in different segments were lower in all models than in previous segments except two structural breaks in 2010, which are characterized by higher values of the intercepts (Czech nationals and Czech nationals organized crime time series) (see Table 2).

## 4. Discussion

The analysis identified structural breaks in most of the methamphetamine-related time series in the period 2009–2016. This period is related to the tightening of methamphetamine precursor regulation and accompanying processes at the methamphetamine market and in methamphetamine supply (involvement of organized crime groups and foreign nationals). The intercepts in the segments of the model were in most cases higher than in the previous ones, suggesting that the regulation led to an increase in the drug supply and nonfatal intoxications. However, to understand the influence of the tightened regulation over sales of the medications containing pseudoephedrine it is significant to focus on the specific categories of time series and also interpret the results taking into account other potential influences on the time series, cf. [40], and control time series, cf. [35]. The control heroin time series showed that the majority of segments were characterized by a lower value of intercepts than in the previous segments, while the methamphetamine related series showed higher values. These different results support the findings of the analysis, pointing to the fact that no underlying mechanism influenced both time series.

One of the structural breaks in the overall methamphetamine arrests time series was identified in the period of interest in 2013 (2013/2, cf. 2012/8, 2013/5) and one in the methamphetamine nonfatal intoxications time series. This points to the fact that at first, the 2009 regulation that tightened control over sales of the medications containing pseudoephedrine did not have an immediate impact on the methamphetamine related arrests and nonfatal intoxications, which is line with the body of literature suggesting a decrease or no effect of the methamphetamine precursor regulation on the methamphetamine arrests time series [15,16,17] and hospital admissions [20,21]. Furthermore, our results are line with the finding that the regulations focused on small-scale producers reportedly had little effect on hospital admissions [21]. The 2013 structural breaks might be explained by the development at the methamphetamine market, gradually higher involvement of organized crime and foreign nationals. However, 2013 is also characterized by a change in the police priorities (inclusion of drug market policing among priorities) that plausibly increased the overall number of arrests and nonfatal intoxications [31,32,41]. The Czech nationals time series showing a development similar to the overall methamphetamine series supports the idea that the overall number of arrests was not caused by foreign nationals. Thus, based on previous research [31,32] and on the results of this analysis we assume that in 2013 these time series were strongly influenced by the change in the Czech police priorities (inclusion of drug market policing among priorities).

In comparison to the general time series, the more specific arrest time series shows a different picture. This is in line with Mazerolle et al. [14], who found that methamphetamine precursor regulation had a different impact on specific time series: decrease in the production of methamphetamine, increase in supply incidents, and no change in possession. We can observe structural breaks and their confidence intervals in the period immediately following the methamphetamine precursor regulations (2009–2010) in the specific methamphetamine time series (foreign nationals, Vietnamese nationals), specific precursor time series (neighboring countries nationals, foreign nationals, Vietnamese nationals), and organized crime time series (overall, Czech, foreign and Vietnamese nationals). The methamphetamine Vietnamese nationals time series shows a similar development as the foreign nationals time series because a notable proportion of the foreign arrests are Vietnamese nationals, cf. number of cases in the time series in Appendix B, Figure A28 and Figure A29. The increased involvement of foreign nationals and of organized groups is in line with the body of literature. The research showed that regulation might be undermined by an existence of alternative sources of precursors [7], for example the import of precursors from neighboring countries [7,17], which leads to the involvement of organized crime and foreign crime groups [18,19].

Structural breaks were identified at different time points in methamphetamine-related time series, showing the dynamic of the methamphetamine market and supply in this period. First structural breaks are identified in 2008 in overall and Czech nationals organized crime time series. The increase before 2008 might seem peculiar; however, it is explained by the fact that Czech methamphetamine producers reacted to the expected changes in the regulation in advance and were increasing their activity before the regulation was put into practice, cf. NDH [42]. The segment of overall and Czech nationals organized crime time series starting in 2008 ended in 2010 and was followed by the segment with a lower value of intercept. Interestingly, the only segments identified in the heroin control series that showed higher values of intercepts were in Czech nationals and Czech national organized crime started in 2010, suggesting that the Czech nationals switched to the heroin market.

The involvement of foreign nationals at the methamphetamine market and involvement of organized groups was gradual, as the previous study suggested [29]. The first signs of foreign nationals involvement are observed almost immediately after the 2009 regulations on the structural breaks in neighboring countries nationals precursor time series, foreign nationals and Vietnamese nationals methamphetamine time series. These are followed later on by the next structural breaks and an increase in the intercepts of segments in foreign nationals and Vietnamese nationals methamphetamine, precursor, and organized crime time series. Although the structural breaks are placed between 2012 and 2014, they have confidence intervals covering longer periods and we can observe some changes in the time series around 2010. Therefore, it is plausible to assume that this increase was not caused only by the change in police priorities in 2013; however, it might have strengthened the increase after 2013.

The strength of the study is that it provides further insight into the effectiveness of the methamphetamine precursor regulation, investigating the regulation in a new context. Furthermore, the study uses a relatively detailed data set provided by the NDH, which allowed for the investigation of the nationality and organized crime involvement, which had not been investigated statistically in studies of this topic. The strength of the study is also the use of the structural break approach to the analysis, which allowed inferring the changes in the time series endogenously from the data. This study also fills the research gap identified by the systematic review [7], because it was focused on the methamphetamine precursor regulation in the European context, using statistical methods.

The limitation of the study is that the missing data had to be imputed, which might have influenced the analysis. However, the influence of this is limited to one year and the development of the time series in this year was estimated based on the rest of the time series, which should provide appropriate estimates. Furthermore, this analysis is limited by its ecological nature; however, the other data, such as official reports reviewed by Petruželka [29], show similar findings, thus triangulating the results. Other factors that might have influenced the time series are discussed in this article based on previous research [31,32,41]. The significant factor is change in the police priorities in 2013 (inclusion of drug market policing among priorities) and corresponding increase in the police activity at the drug market [31,32,41]. The effect of this change was discussed in the respective sections of the discussion. Furthermore, to address the limits, we used control time series. Another limit of the study is that it is based on administrative police data, which have a number of limitations themselves, such as the accuracy of reporting and police discretion in recording incidents [31,43,44]. Furthermore, the analysis did not take into account other metrics, such as price and purity data, which are not available in the Czech Republic in a form allowing for the use of these metrics in this kind of time series analysis.

## 5. Conclusions

This study provides an insight into the impact of methamphetamine precursor regulation, which is considered to be one of the most important tools of supply reduction and a tool with potential public health impact. The general rate of methamphetamine arrests and nonfatal intoxications plausibly did not increase in relation to the introduction of regulation; however, the increasing involvement of organized crime and foreign nationals at the methamphetamine and precursor market is apparent. Methamphetamine precursor regulation at a well-established drug market characterized by small-scale production might lead to a proliferation of international and organized crime groups and no change in the overall number of arrests. These changes of drug supply towards more organized groups could have many consequences because their presence imposes significant costs for the society in many different areas both nationally and internationally. Furthermore, the study shows that methamphetamine precursor regulation is not necessarily effective in suppressing the methamphetamine drug market and public health indicators, such as methamphetamine nonfatal intoxications.

## Figures and Tables

**Table 1:** The structural breaks identified in time series.

Specific Time Series	Number of Breaks	Date of Breakpoint	cf.		Date of Breakpoint	cf.		Date of Breakpoint	cf.		Date of Breakpoint	cf.	
			Lower	Upper		Lower	Upper		Lower	Upper		Lower	Upper
Methamphetamine Arrests													
Overall	2	2006/2	2004/11	2007/2	2013/2	2012/8	2013/5						
Czech nationals	1	2013/2	2012/8	2013/7									
Foreign nationals	1	2009/6	2008/5	2009/9	2013/1	2011/12	2013/4						
Vietnamese nationals	1	2010/3	2009/6	2010/4	2012/12	2009/2	2013/10						
Neighboring countries nationals	1	2012/10	2011/10	2013/1									
Precursor Arrests													
Overall	1	2011/12	2011/7	2012/1									
Czech nationals	1	2011/12	2011/7	2012/1									
Foreign nationals	1	2013/10	2009/8	2003/12									
Vietnamese nationals	1	2013/4	2007/9	2013/8									
Neighboring countries nationals	1	2009/5	2005/6	2009/6									
Methamphetamine and Precursor-Related Organized Crime Arrests													
Overall	4	2006/3	2004/11	2007/5	2008/2	2007/10	2008/6	2010/1	2009/12	2010/4	2014/1	2011/8	2014/5
Czech nationals	3	2006/3	2005/3	2007/2	2008/2	2007/11	2008/5	2010/1	2009/12	2010/4			
Foreign nationals	1	2013/9	2010/6	2014/2									
Vietnamese nationals	1	2013/1	2009/6	2013/4									
Neighboring countries nationals	0												
Heroin Arrests													
Overall	1	2010/5	2010/2	2011/2									
Czech nationals	2	2007/12	2007/2	2009/10	2010/5	2010/3	2010/7						
Foreign nationals	1	2011/10	2011/4	2014/2									
Vietnamese nationals	1	2011/10	2011/9	2014/1									
Neighboring countries nationals	0												
Heroin-Related Organized Crime Arrests													
Overall	1	2009/6	2009/5	2011/6									
Czech nationals	2	2008/4	NA	NA	2010/6	NA	NA						
Foreign nationals	1	2009/7	2009/6	2014/6									
Vietnamese nationals	0												
Neighboring countries nationals	0												
Nonfatal Intoxications													
Methamphetamine	1	2013/11	2012/12	2014/4									
Heroin	1	2010/7	2010/2	2011/11									

**Table 2:** The intercepts in the different models and different segments of models.

Time Series	Intercepts					
	Not Segmented Model	Segmented Models				
		Segment 1	Segment 2	Segment 3	Segment 4	Segment 5
Methamphetamine Arrests						
Overall	123.3	94.0	111.5	158.3		
Czech nationals	113.0	100.2	139.5			
Foreign nationals	8.3	3.3	7.8	14.9		
Vietnam nationals	4.4	0.9	5.2	8.5		
Neighboring country nationals	2.0	1.3	3.5			
Precursor Arrests						
Overall	7.2	2.2	14.3			
Czech nationals	6.4	1.8	13.0			
Foreign nationals	0.6	0.3	1.4			
Vietnamese nationals	0.1	0.0	0.3			
Neighboring countries nationals	0.2	0.0	0.3			
Methamphetamine and Precursor-Related Organized Crime Arrests						
Overall	9.7	15.1	7.9	28.1	3.8	9.2
Czech nationals	8.0	14.1	6.9	26.6	3.8	
Foreign nationals	1.7	1.1	3.4			
Vietnamese nationals	1.2	0.6	2.4			
Neighboring countries nationals	NA					
Heroin Arrests						
Overall	8.5	12.3	5.4			
Czech nationals	5.9	8.1	10.8	3.4		
Foreign nationals	2.4	3.3	1.2			
Vietnamese nationals	1.0	1.5	0.2			
Neighboring countries nationals	NA					
Heroin-Related Organized Crime Arrests						
Overall	1.9	3.9	0.4			
Czech nationals	1.1	1.7	3.9	0.1		
Foreign nationals	0.8	1.7	0.2			
Vietnamese nationals	NA					
Neighboring countries nationals	NA					
Nonfatal Intoxications						
Methamphetamine	2.7	2.2	4.4			
Heroin	1.7	2.4	1.0			

**Figure A1:**
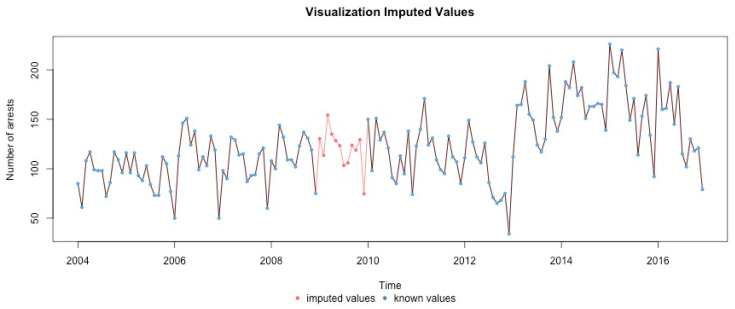
Methamphetamine arrests 2004–2016 in the Czech Republic—imputed missing values.

**Figure A2:**
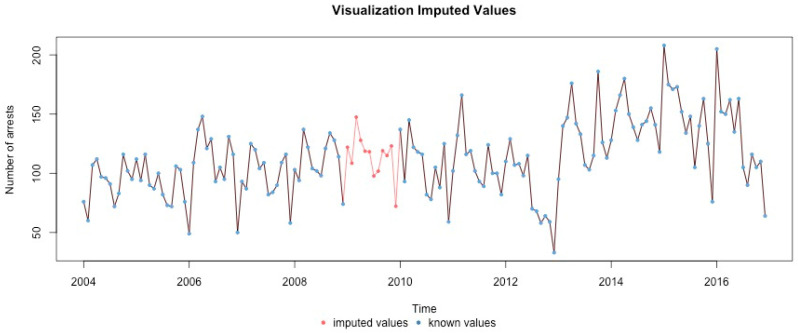
Methamphetamine Czech nationals arrests 2004–2016 in the Czech Republic—imputed missing values.

**Figure A3:**
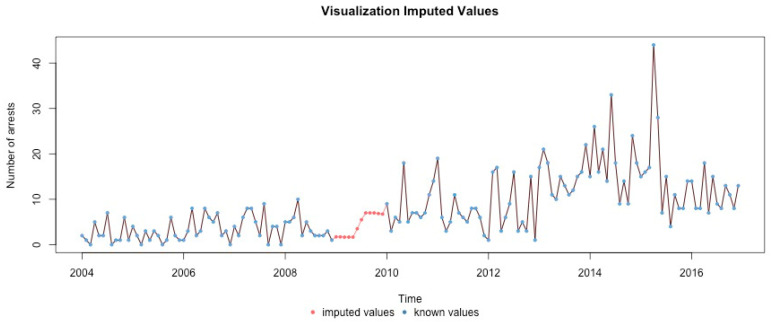
Methamphetamine foreign nationals arrests 2004–2016 in the Czech Republic—imputed missing values.

**Figure A4:**
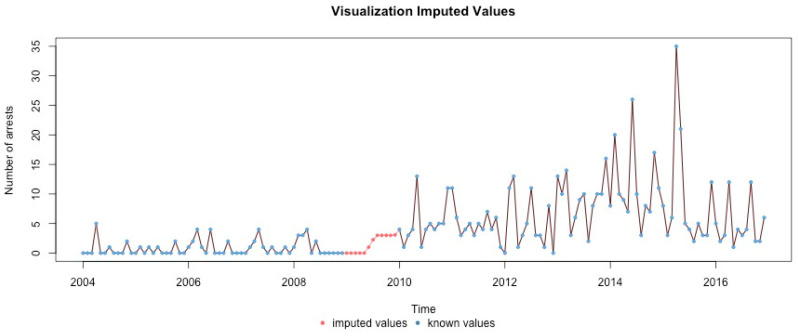
Methamphetamine Vietnamese nationals arrests 2004–2016 in the Czech Republic—imputed missing values.

**Figure A5:**
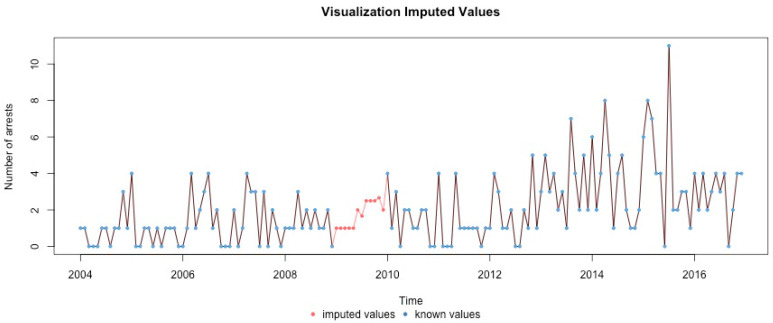
Methamphetamine neighboring countries arrests 2004–2016 in the Czech Republic—imputed missing values.

**Figure A6:**
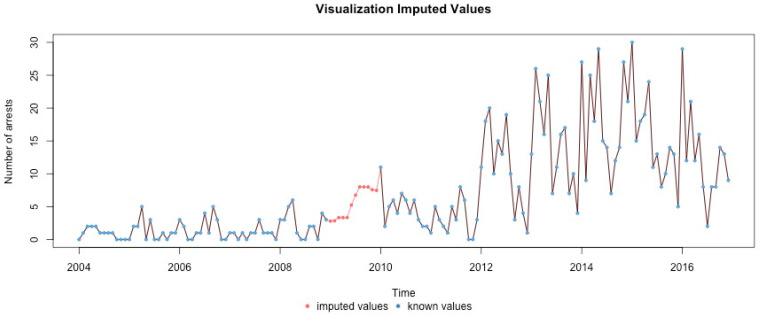
Precursor arrests 2004–2016 in the Czech Republic—imputed missing values.

**Figure A7:**
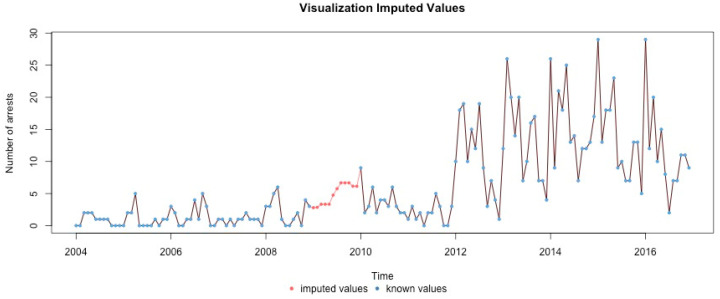
Precursor Czech nationals arrests 2004–2016 in the Czech Republic—imputed missing values.

**Figure A8:**
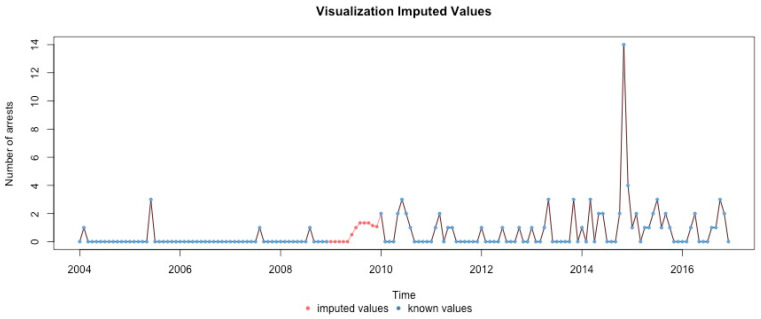
Precursor foreign nationals arrests 2004–2016 in the Czech Republic—imputed missing values.

**Figure A9:**
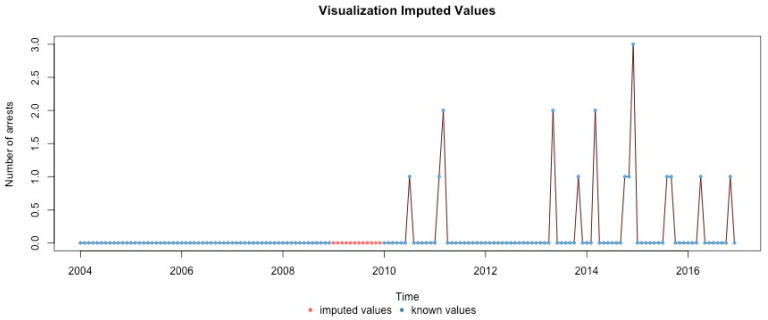
Precursor Vietnam nationals arrests 2004–2016 in the Czech Republic—imputed missing values.

**Figure A10:**
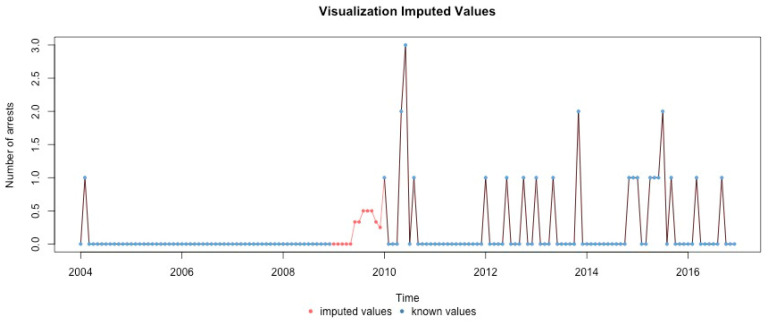
Precursor neighboring countries nationals arrests 2004–2016 in the Czech Republic—imputed missing values.

**Figure A11:**
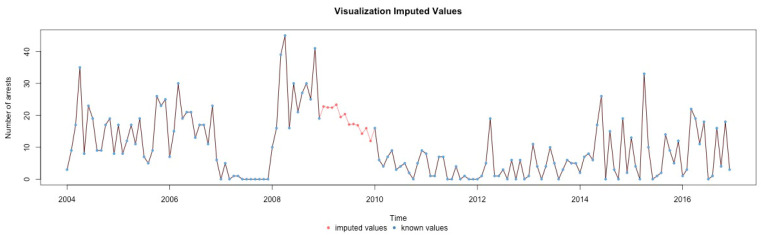
Methamphetamine-related organized crime arrests 2004–2016 in the Czech Republic—imputed missing values.

**Figure A12:**
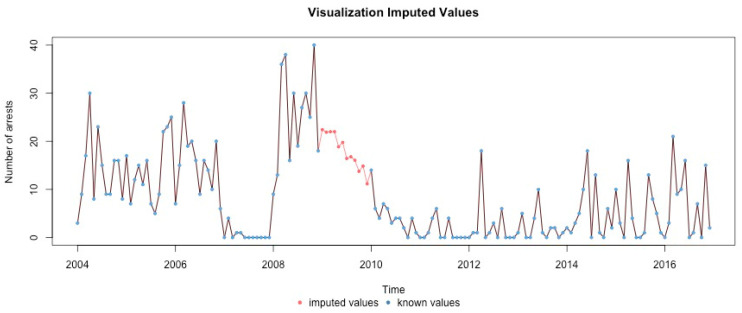
Methamphetamine-related organized Czech nationals crime arrests 2004–2016 in the Czech Republic—imputed missing values.

**Figure A13:**
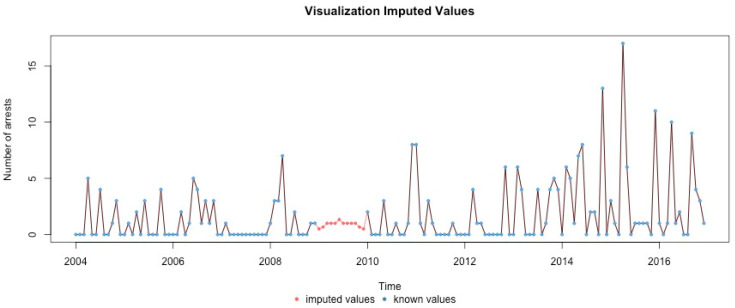
Methamphetamine-related organized foreign nationals crime arrests 2004–2016 in the Czech Republic—imputed missing values.

**Figure A14:**
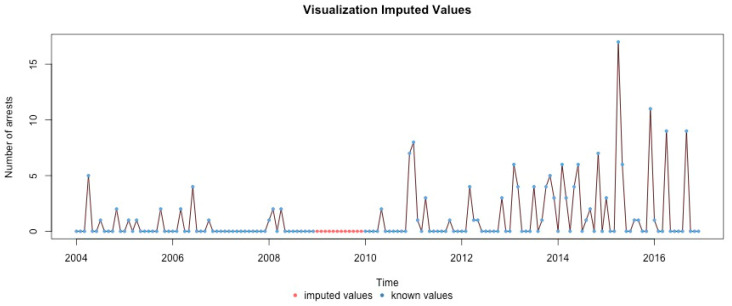
Methamphetamine-related organized Vietnamese nationals crime arrests 2004–2016 in the Czech Republic—imputed missing values.

**Figure A15:**
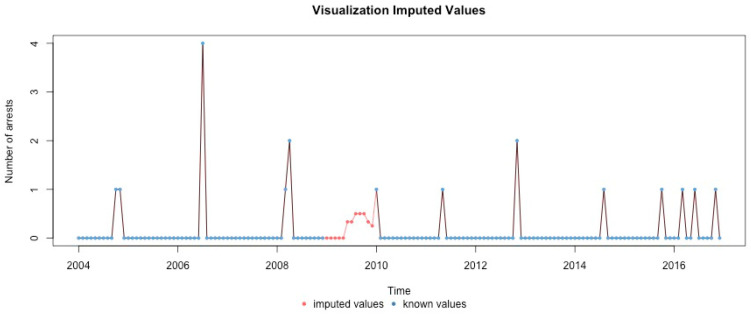
Methamphetamine-related organized neighboring countries nationals crime arrests 2004–2016 in the Czech Republic—imputed missing values.

**Figure A16:**
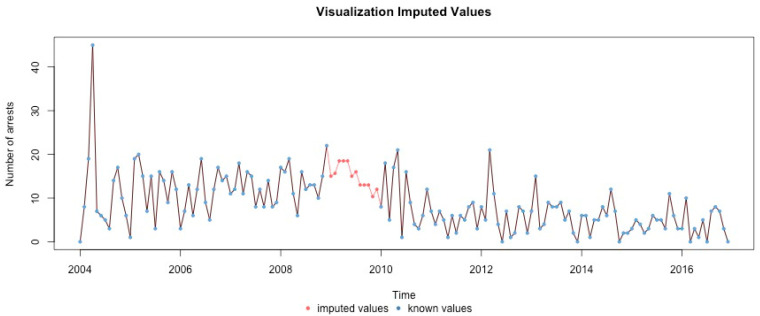
Heroin arrests 2004–2016 in the Czech Republic—imputed missing values.

**Figure A17:**
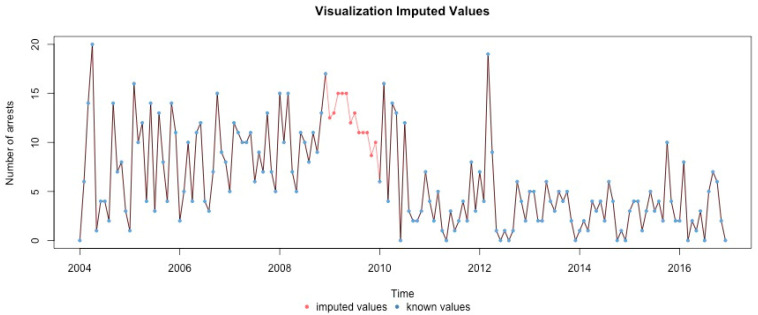
Heroin Czech nationals arrests 2004–2016 in the Czech Republic—imputed missing values.

**Figure A18:**
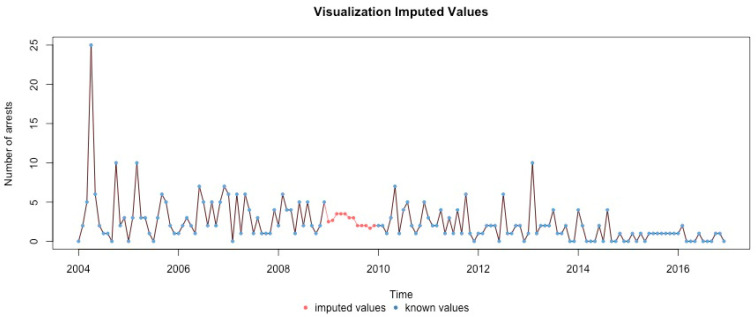
Heroin foreign nationals arrests 2004–2016 in the Czech Republic—imputed missing values.

**Figure A19:**
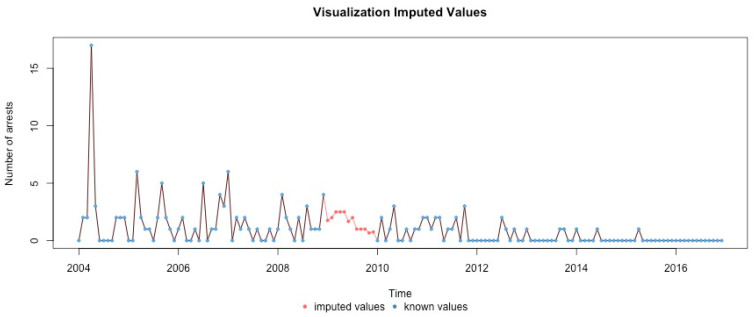
Heroin Vietnam nationals arrests 2004–2016 in the Czech Republic—imputed missing values.

**Figure A20:**
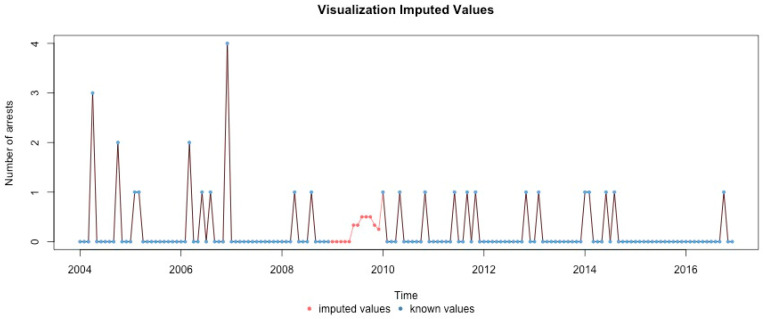
Heroin neighboring country nationals arrests 2004–2016 in the Czech Republic—imputed missing values.

**Figure A21:**
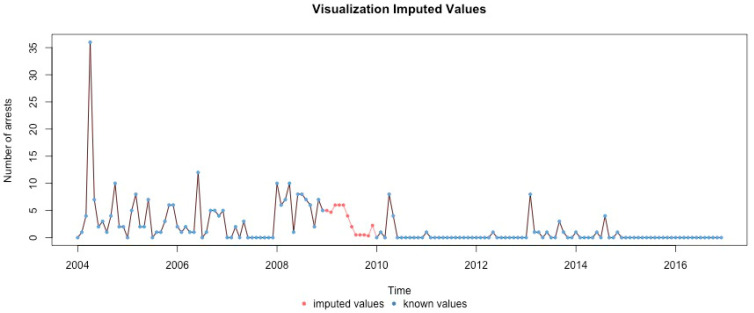
Heroin organized crime arrests 2004–2016 in the Czech Republic—imputed missing values.

**Figure A22:**
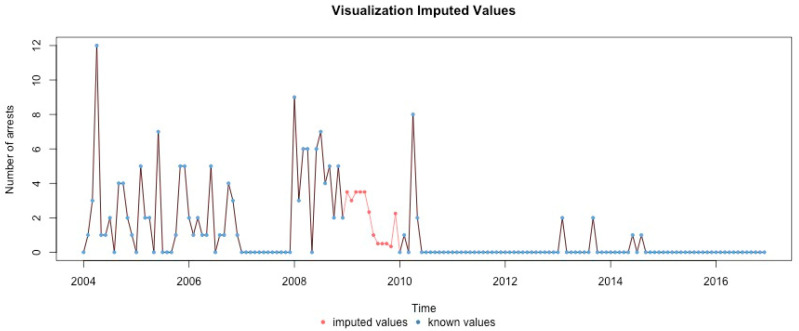
Heroin organized Czech nationals crime arrests 2004–2016 in the Czech Republic—imputed missing values.

**Figure A23:**
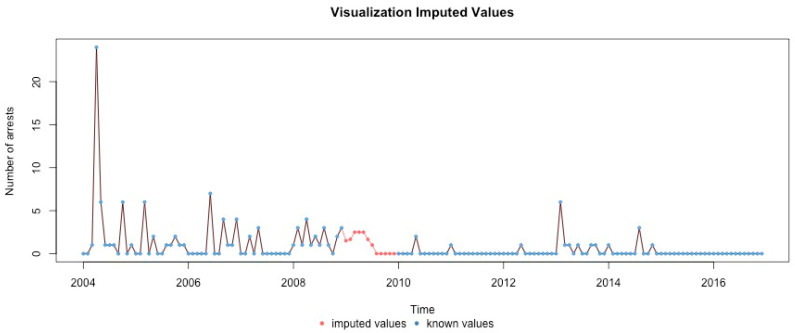
Heroin organized foreign nationals crime arrests 2004–2016 in the Czech Republic—imputed missing values.

**Figure A24:**
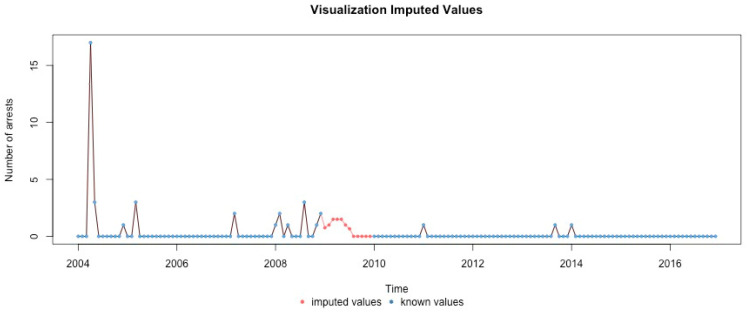
Heroin organized Vietnamese nationals crime arrests 2004–2016 in the Czech Republic—imputed missing values.

**Figure A25:**
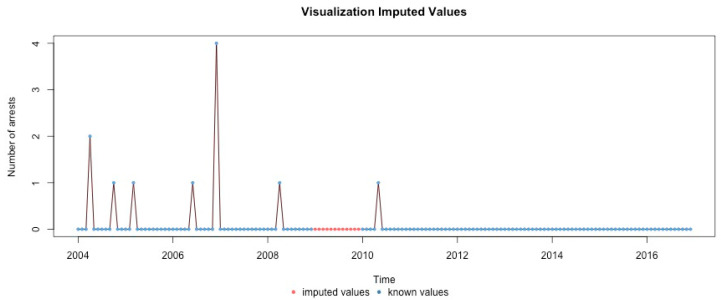
Heroin organized neighboring countries nationals crime arrests 2004–2016 in the Czech Republic—imputed missing values.
